# *Borrelia* BBJ25 is a plasmid-encoded Omp85-family protein associated with a potential export system

**DOI:** 10.1042/BSR20254082

**Published:** 2026-02-06

**Authors:** Abigail Nixon, Evelina Soliman, Jaroslaw Bryk, Martin Carr, Christopher D. O. Cooper, Richard J. Bingham

**Affiliations:** Department of Physical and Life Sciences, University of Huddersfield, School of Applied Sciences, Huddersfield, England, HD1 3DH, U.K.

**Keywords:** β-barrel, BBJ25, Borrelia, lipoprotein export, plasmids

## Abstract

The *Borrelia* genome consists of a linear chromosome and numerous linear and circular plasmids. Using a computational framework, we examined the plasmid proteome of *Borrelia burgdorferi sensu lato* to identify potential outer membrane (OM) β-barrel proteins. Our approach identified BBJ25 on plasmid lp38 as an Omp85 superfamily domain with structural homology to BamA/TamA. Analysis of the surrounding genes reveal a cluster of seven genes. Structure–function analysis (AlphaFold3 and DALI) points towards a role in membrane transport, with two chaperone proteins (BBJ23, BBJ24), the Omp85-family domain BBJ25, a homodimeric MacB-like type-VII ABC transporter with an associated ATP-binding domain (BBJ26, BBJ27), a LolA-like domain (BBJ28), and one protein of unknown function (BBJ29). Taken together, this points towards a role in the ATP-driven extraction of a non-polar ligand, possibly lipoproteins, from the inner membrane and delivery to the OM. Structural homology is detected to *Escherichia coli* proteins involved in lipoprotein sorting (LolACDE). Wider analysis of the *Borrelia* genus revealed two major variants of this 7-gene cluster, distributed across multiple different linear elements including lp17, lp28, lp38, and the linear chromosome of *Borrelia turcica*. *Borrelia valaisiana* was found to have both allelic variants coexisting in the same genospecies on different linear plasmids (lp28-3 and lp28-8). Previous studies have reported up-regulation of these genes in response to mammalian signals. The conservation of these proteins throughout the *Borrelia* genus, coupled with the occurrence of multiple copies in some genospecies, point towards a critical function. The identity of the target ligand remains uncertain and requires experimental verification.

## Introduction

The *Borrelia burgdorferi sensu lato* complex contains numerous species, all with an obligate parasitic lifestyle infecting a range of vertebrates and transmitted by ticks of the *Ixodes* genus. Several genospecies of *Borrelia* are able to infect humans via zoonotic transmission causing either Lyme disease (LD) or relapsing fever (RF) [1]. The structure of the *Borrelia* genome is unusual in that it is comprised of a linear chromosome of 0.9 Mbp and a large, but variable complement of both linear and circular plasmids making up another 0.4–0.65 Mbp [2]. The loss of individual plasmids is frequently associated with reduced infectivity in mice and/or reduced survival within the tick vector [3–5]. Many of the plasmids are universally present in natural samples and encode a range of proteins that are important for virulence and pathogenesis such as the surface lipoproteins OspA/B/C, DbpA/B, and BBK32 [6].

The protein component of the *Borrelia* outer membrane (OM) is distinct from other Gram-negative diderm bacteria in that it includes over 100 different lipoproteins and comparatively few integral OM-proteins [6]. In other Gram-negative bacteria, it is estimated that between 1.5% and 2.4% of all proteins are OM β-barrels [7]. Based on this range, from the 1291 proteins in *B. burgdorferi B31*, 19–31 OM β-barrels might be expected, whereas less than ten have been identified [6,8–10]. Previous efforts to identify novel OM β-barrels have focussed on the linear chromosome [9]. However, the many circular and linear plasmids have so far remained unexplored for potential OM β-barrel sequences. Therefore, we conducted a comprehensive search of the plasmid components of all available (*n* = 21) non-redundant *Borrelia* genomes. The results reveal a single plasmid-encoded OM β-barrel, BBJ25, present in all *Borrelia* genomes investigated. BBJ25 forms part of a 7-gene cluster that occurs on a range of different plasmids and may be involved in OM biogenesis.

Our current understanding of *Borrelia* OM biogenesis consists of three major systems: the barrel assembly machinery (BAM) responsible for the export and insertion of OM β-barrel proteins [11], the Lpt system, which, in the absence of lipopolysaccharides, is likely responsible for the transport of glycolipids [12], and finally, there is evidence for an incomplete localisation of lipoprotein (Lol) pathway [13]. The *Escherichia coli* Lol system consists of the LolCDE complex (a heterodimeric ATP-binding cassette (ABC) transporter), and two structurally homologous transporters, LolA (a periplasmic chaperone) and LolB (the OM receptor, which is itself a lipoprotein) [14]. Only one of these components has been previously characterised in *Borrelia*—the LolA homologue BB0346 [13]. Therefore, the precise export mechanism of lipoprotein export in *Borrelia* remains uncertain with knockout studies reporting conflicting results [12,15]. A component of the *Borrelia* Lpt system, BbLptD, has been shown to be responsible for flipping surface lipoproteins into the outer leaftlet [15], while Bowen *et al.* reported normal surface exposure of the lipoprotein CspA after down-regulation of BbLptD [12]. Cross-talk between these pathways may occur, as in other Gram-negative bacteria, where chaperone proteins may shuttle components such as lipoproteins from one system to another [16].

The combination of structure prediction using AlphaFold3 with subsequent analysis by the distance matrix alignment (DALI) algorithm provides a powerful method of predicting function and detecting remote homologies [17]. Here, we present such an analysis of proteins BBJ23-29, revealing structural homology to the Lol-sorting machinery of *E. coli*. We propose that *BBJ23-29* encode for an ABC transporter (permease and ATP-binding domains), three periplasmic carrier proteins, and BBJ25—an Omp85-family domain that may facilitate delivery of a non-polar ligand to the OM or extracellular space. The precise identity of the non-polar ligand(s) remains unclear. The similarity to the *E. coli* Lol-sorting machinery is intriguing, as the identification of a complete Lol-sorting machinery in *Borrelia* would enhance our understanding of OM biogenesis and, because many surface lipoproteins are involved in pathogenicity, it may provide a target for the development of novel antimicrobials.

## Hypothesis

We hypothesise that the plasmid component of the *Borrelia* genus may encode one or more OM proteins with a β-barrel topology. Confirming their presence would enable targeted experiments, enhancing our understanding of OM biogenesis and plasmid genetics, both of which exhibit features unique to *Borrelia*.

## Results

### A computational framework to detect plasmid encoded OM β-barrels

**Figure 1:**
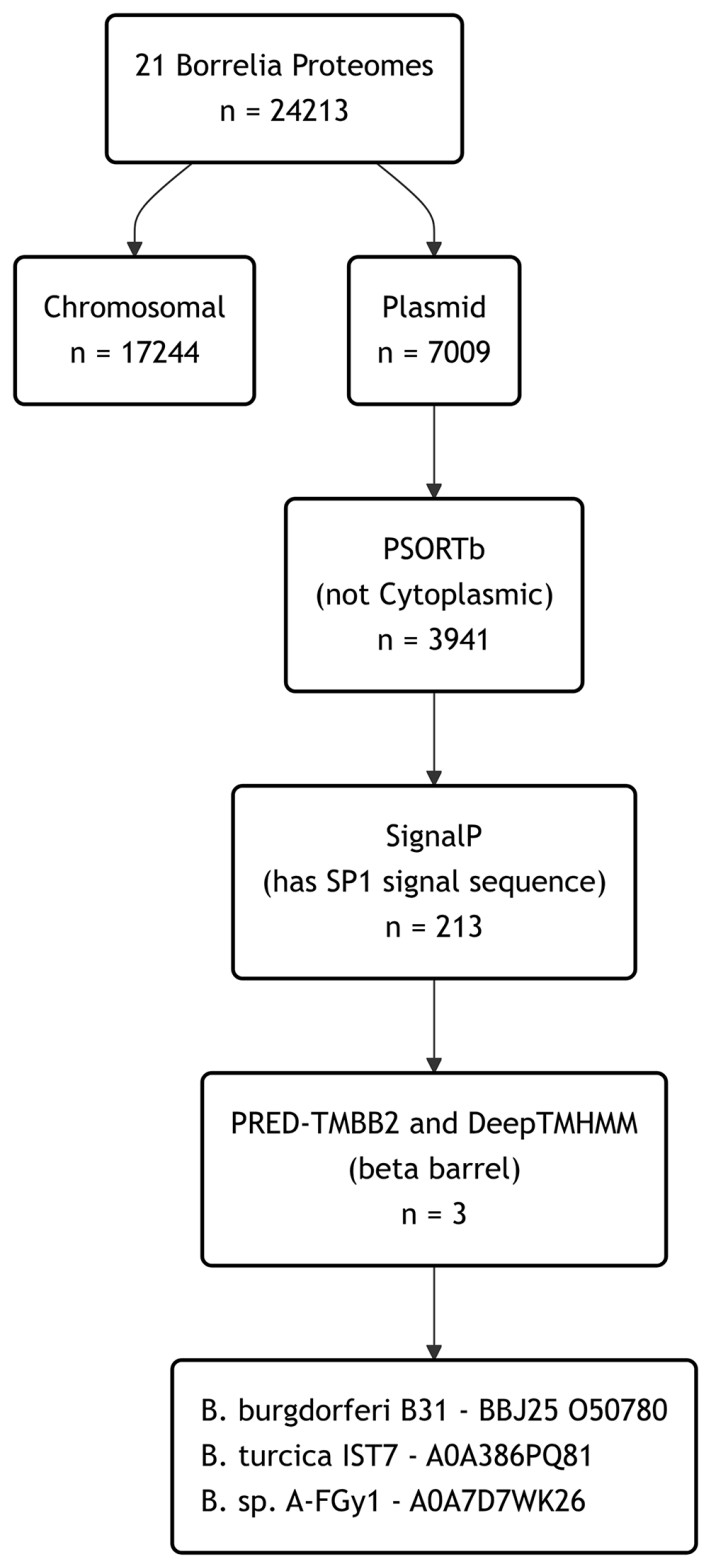
Hierarchical framework to predict OM β-barrels in the complete plasmid proteome of *Borrelia* The number of protein sequences retained at each stage is indicated. UniProtKB accession numbers are given.

Historically, predicting OM β-barrel proteins from sequence data was a significant challenge and required hierarchical computational frameworks with up to 14 steps. Such methods have been successful in predicting OM β-barrels from both *Treponema* [18] and from the linear chromosome of *B. burgdorferi sensu stricto* [9]. In the last decade, prediction methods have improved in both accuracy and scope [19,20] allowing a simpler framework to be employed. Starting from 21 non-redundant *Borrelia* proteomes available at UniProtKB (Supplementary Table S1), plasmid proteome components were extracted, yielding a total of 7009 predicted protein sequences. These sequences were then subjected to a four-step computational framework, where the combined results from all prediction algorithms were used to identify potential plasmid-encoded OM β-barrel proteins (Figure 1).

Firstly, cytoplasmic proteins were predicted using PSORTb, a predictor of bacterial protein subcellular localisation [21]. Although precise, PSORTb can be limited by low coverage—the proportion of proteins receiving a prediction in a proteome at a given level of precision. We found this to be the case for the *Borrelia* plasmid proteome, as only 53% of sequences received a prediction of any type; the remainder (47%) were annotated as ‘unknown’. All proteins with a predicted cytoplasmic location were removed at this stage (3068 sequences removed, 3941 remain).

Any potential OM β-barrel must possess a signal peptide to enable translocation across the cytoplasmic membrane, and subsequent trafficking to the OM. We used SignalP v6.0 [19] to select only those proteins predicted to have a signal peptidase type-1 (SP1) signal sequence, therefore removing any remaining cytoplasmic proteins and all predicted lipoproteins processed by signal peptidase II (3728 sequences removed, 213 remain).

Finally, two predictors of β-barrel topology were then employed, PRED-TMBB2 [22] and DeepTMHMM [20]. This hierarchy identified three proteins, two of which are currently annotated as OM proteins: The first two are orthologous with 84.2% sequence identity: BBJ25 from *B. burgdorferi* B31 (plasmid lp38) and a BamA/TamA family OM protein from *Borrelia* sp. A-FGy1 (plasmid p11AFGy1). The third is a more distantly related uncharacterised protein from *Borrelia turcica* IST7 (plasmid lp35) with 17.6% sequence identity to BBJ25. Models in the AlphaFoldDB resemble a BamA/TamA β-barrel fold for all three proteins, but these proteins are distinct from the chromosomally encoded *Borrelia* BamA (BB0795) [11].

### BBJ25 is part of a conserved cluster of seven genes

**Figure 2:**
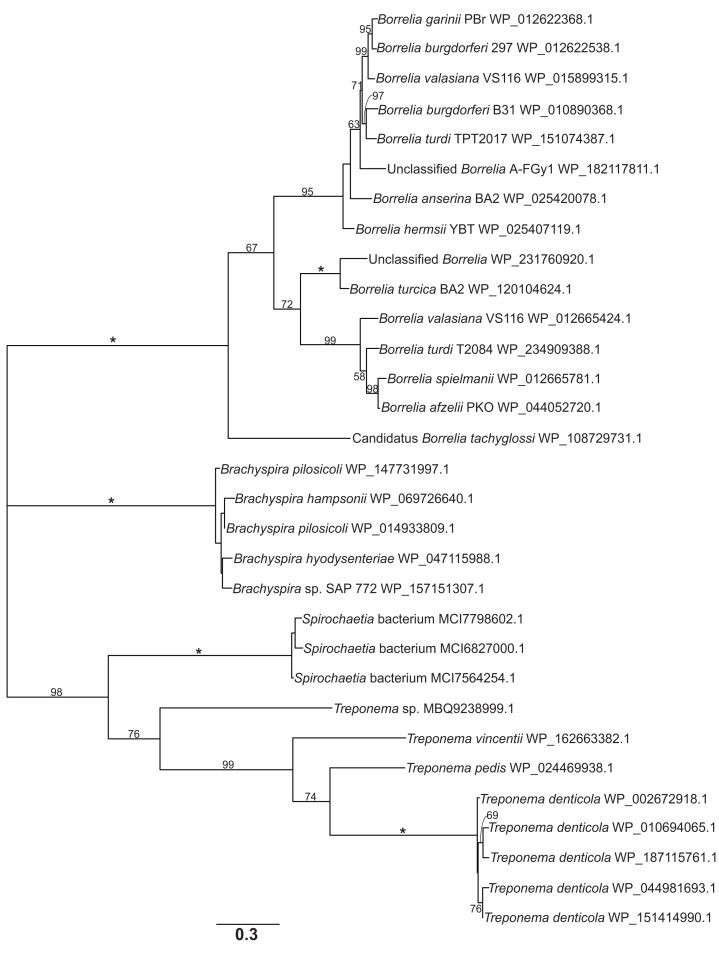
Unrooted maximum likelihood phylogeny of BBJ25 homologues The phylogeny was created in MEGA X from an alignment of 463 amino acid positions using the JTT + G amino acid substitution model. Support values were generated from 1000 bootstrap replicates, with bootstrap support percentages shown above branches. Asterisks denote 100% bootstrap; values <50% are omitted. Branches are drawn proportional to the number of substitutions per site as indicated by the scale bar.

A PSI-BLAST search [23] using BBJ25 as a query sequence revealed homologous sequences from all major families within the phylum of *Spirochaetes* with the exception of *Leptospira*. Phylogenetic analysis demonstrates three distinct groups clustered by spirochete family (Figure 2). Orthologues of BBJ25 were found in *Borrelia* (both LD and RF-type), *Brachyspira*, and *Treponema*. The sequence identity of BBJ25 homologues between the *Brachyspira, Treponema*, and *Borrelia* families is in the region of 20%–24% over the full length of the protein (346 residues) and so are highly divergent.

**Figure 3:**
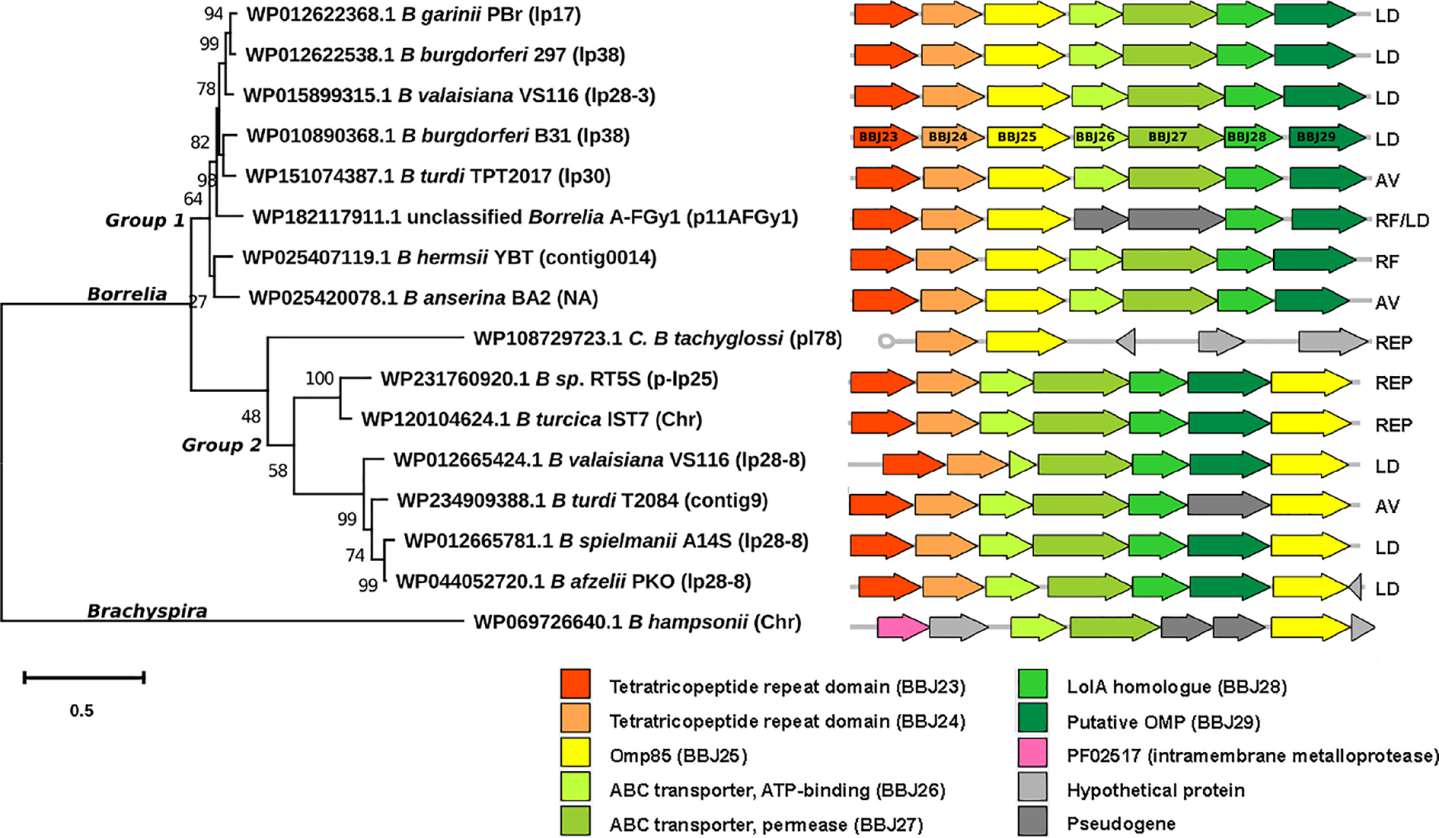
Phylogenetic analysis of BBJ25 protein sequences from *Borrelia* species and *Brachyspira* The phylogenetic tree was generated in MEGAX using the maximum likelihood method and a JTT matrix-based model [34]. A bootstrap method was applied using 1000 replications. Branch lengths are measured in number of substitutions per site. A graphical representation of the seven-gene cluster is shown and the group each species belongs to is indicated as either LD, RF, Avian (AV), or reptile (REP) associated. Graphical representation generated using the gggenes package in R [36] using nucleotide sequence lengths in GenBank (NCBI).

Consistent with the notion that *Borrelia* forms a single genus [24], the clustering within *Borrelia* (Figure 2) was not correlated with either RF/LD-type genospecies and does not match the clustering that would be expected from vertical descent alongside the chromosome. Attempts were made to explain the clustering based on plasmid type, geographical location, and host vectors; however, no correlation could be found. Throughout their history, the linear plasmids of *Borrelia* have undergone many recombination and duplication events, and it is not unusual for any given gene to reside on different linear plasmids in different genospecies [25]. Within *Borrelia*, homologues of BBJ25 were found on a range of different linear plasmids (lp38, lp17, lp28-3, and lp28-8), therefore it was of interest to investigate the physical location of BBJ25 within each plasmid. Analysis of the surrounding genes revealed two distinct groups, clustering separately in the phylogenetic analysis (Group 1 and Group 2; Figure 3). Within both groups, there is a seven-gene cluster, which, based on existing annotations, consists of two tetratricopeptide repeat (TPR) domain proteins (BBJ23-24), an ABC transporter with both ATP-binding (BBJ26) and permease domains (BBJ27), the BamA/TamA homologue BBJ25, an OM lipoprotein-sorting protein (BBJ28), and one uncharacterised protein (BBJ29). The gene order is conserved except for BBJ25, where this is found in either the third or last position in the cluster.

In both groups, BBJ25 occupies a position immediately upstream or downstream of an ABC transporter. In the distantly related *Brachyspira*, the order of chromosomal genes is similar to Group 2; two ABC transporter genes (ATP-binding and permease protein) and the BBJ25 homologue are co-linear but are separated by two pseudogenes. The gene structure in *Treponema* genospecies appears to be quite different and the BBJ25 homologues are surrounded by unrelated genes (results not shown).

To test whether the ancestral history of the *Borrelia* BBJ25 homologues (Figure 3) is consistent with the surrounding region, the nucleotide sequences of the whole seven-gene cluster were aligned, and the evolutionary history was inferred based on maximum likelihood methods. This recovered a similar tree with all major branch points occurring in the same order (Supplementary Figure S1A). Furthermore, realigning the nucleotide sequences omitting the coding sequence for all BBJ25 homologues produced a tree with identical branching (Supplementary Figure S1B).

### Putative functional annotation of BBJ23-29

**Figure 4:**
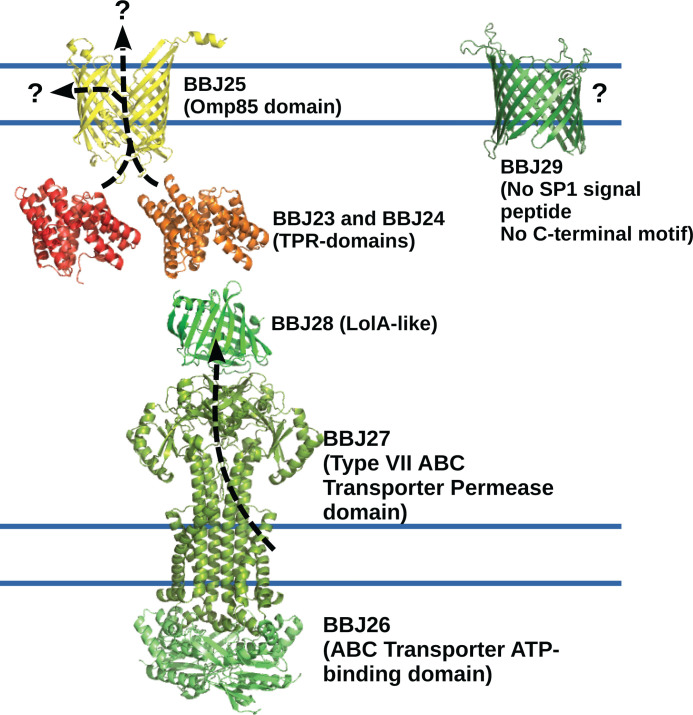
Proposed model of ligand export by BBJ23-29 BBJ26 and BBJ27 are proposed to be analogous to the LolCDE transporter in *E. coli* acting as an exporter for a non-polar ligand. This ligand is then received by the LolA homologue BBJ28. The TPR-repeat contain proteins BBJ24 and BBJ28 may act as chaperones guiding the non-polar ligand to the Omp85-family protein BBJ25. The role of BBJ29 is unknown.

DALI is now routinely used to compare predicted 3D structures with those in the Protein Data Bank to assign a putative function [17]. AlphaFold3 models of proteins BBJ23-29 were analysed using the DALI algorithm [26]. The predicted functions are self-consistent, all relating to the export of a non-polar ligand through the inner membrane (IM) and delivery to the OM (Figure 4).

BBJ23 and BBJ24 are remote homologues with 22% sequence identity and are structurally homologous to TPR-containing proteins. The AlphaFold3 models reveal a structure with 11 helices that form 5 TPR units (Figure 4). The top DALI hits for BBJ23 and BBJ24 are all TPR-containing proteins involved in the transport of non-polar ligands such as sterols, phospholipids or lipoprotein, many of which are also involved in forming oligomers (Supplementary Table S2). Both BBJ23 and BBJ24 have an SP1-type signal peptide as predicted by SignalP, but are not predicted to be membrane proteins, therefore they may act as periplasmic chaperone proteins.

Consistent with the existing annotation, results from the DALI analysis indicate that BBJ26 forms a dimeric ATP-binding domain that associates with a homodimer of the membrane-spanning permease domain BBJ27. Based on this prediction, an AlphaFold3 model of the complete ABC transporter was generated with two copies of each polypeptide (Figure 4). BBJ27 is predicted to have 4 TM helices per monomer with strong structural homology to a type VII ABC transporter, a type that is associated with export processes [27]. The top hit from the DALI search was the *E. coli* ABC transporter LolE [28]. In *E. coli*, LolD and LolE form a heterodimeric permease domain that extracts lipoproteins from the IM and shuttles them to the soluble periplasmic chaperone protein—LolA. BBJ28 was found to be a structural homologue of this lipoprotein carrier, the three top hits in DALI were all annotated as Lipoprotein localisation factor (LolA-like) (Supplementary Table S2). The AlphaFold3 model of BBJ28 (Figure 4 and Supplementary Figure S2) is structurally similar to the apo-form of *E. coli* LolA, both having an unclosed β-barrel forming a non-polar central cavity which is capped by a short alpha-helix. In *E. coli*, this helix is displaced by the N-terminal triacyl chains of a lipoprotein as it is carried across the periplasm before being passed on to the OM lipoprotein LolB [29]. To date, no homologue of LolB has been identified in *Borrelia*, so the mechanism of lipoprotein insertion into the OM may be different.

The AlphaFold3 model of BBJ25 reveals an Omp85-type domain (Figure 4). DALI analysis of BBJ25 strongly suggests involvement in the process of protein insertion in the OM, with the top three hits being the mitochondrial sorting and assembly machinery (SAM), the bacterial translocation and assembly module (TamA) and the barrel assembly machinery BamA (Supplementary Table S2). SAM is responsible for the correct insertion of β-barrels into the OM of mitochondria so will not be considered further. In *Borrelia*, OM proteins are already known to be processed by the chromosomally encoded BamA system (BB0795) [11]. It is perhaps unlikely that *Borrelia* would have two parallel systems, with BBJ25 processing OM β-barrels alongside BamA, as there are so few OM β-barrels in the *Borrelia* genome. Furthermore, translocation of OM β-barrels across the periplasm requires the polypeptide-transport-associated (POTRA) domain to act as a critical chaperone [30]. Our results failed to find any POTRA domains in this system. Based on the absence of POTRA domains, we have denoted BBJ25 as an Omp85 superfamily domain rather than BamA or TamA.

Taken together, the function of each of these proteins provides a plausible mechanism to extract a non-polar ligand from the IM and deliver it to the OM or extracellular space.

## Methods

### Identification of putative OM β-barrels encoded by plasmid genes in *Borrelia*

A modified computational framework based on previous studies [9,18] was used to predict putative OM β-barrel proteins encoded within the numerous plasmids of *B. burgdorferi sensu lato*. Plasmid proteomes (Supplementary Table S1) were obtained from UniProt and were subjected to analysis by a series of prediction algorithms as described below. Results from each algorithm were parsed and analysed by a custom R-script to filter sequences, removing them from further analysis based on the results of these predictions. Firstly, sequences predicted to be either ‘Cytoplasmic’ or ‘CytoplasmicMembrane’ by PSORTb 3.0 [21] were eliminated. SignalP 6.0 [19] was used to predict signal peptidase I-type signal sequences. Only those proteins with an ‘SP’ prediction were retained. β-barrel topology prediction was in two stages. Only those proteins with a β-barrel score >0.25 from PRED-TMBB2 [22] and a ‘BETA’ prediction from DeepTMHMM (version 1.0.24) [20] were retained. Sequences annotated as fragments were removed. The raw data and R-code are available at https://github.com/RJBingham/Nixon_2025 [31]. Protein sequences were submitted to the AlphaFold3 server for structure prediction [32] and the top scoring models were analysed by DALI [26].

### Evolutionary analysis

Using BBJ25 (O50780_BORBU, *B. burgdorferi* B31) as a query sequence, two iterations of PSI-BLAST [23] were used to search against all non-redundant protein sequences. From a total of 131 hits, 106 sequences were selected by filtering for query coverage >90% and *E*-value threshold <10^−7^. Redundancy was decreased further by removal of sequences with greater than 95% identity to any other sequence. The resultant 31 sequences were then aligned using MUSCLE using default parameters [33] and a phylogenetic tree was generated using maximum likelihood method and a JTT + G matrix-based model in MEGA X [34]. The JTT + G model was chosen based on a ranking of 60 models by prottest3 [35]. The bootstrap consensus tree was inferred from 1000 replicates. Initial trees for the heuristic search were obtained automatically by applying Neighbor-Join and BioNJ algorithms to a matrix of pairwise distances estimated using the JTT model and then selecting the topology with superior log likelihood value. Neighbouring genes and their locations were identified from complete genome sequences in GenBank (NCBI) and displayed using the gggenes package in R [36].

## Discussion

To date, only nine OM β-barrels have been identified in *Borrelia* (BB0027, BB0142, BB0405, BB0406, BB0418, BB0562, BB0603, BB0795, BB0838), all of which are encoded by chromosomal genes [8–11,37–39]. Here, using a computational framework, we have identified a plasmid-encoded protein, BBJ25, which is predicted to be an Omp85-type OM β-barrel and forms part of a conserved seven-gene cluster, BBJ23–BBJ29. Functional predictions suggest a role in export of a non-polar ligand. The linear plasmids of *Borrelia* harbour numerous pseudogenes alongside genes encoding critical virulence factors whose expression is tightly regulated across the host–vector transmission cycle. It was therefore of interest to investigate the expression of this locus.

Because adaptation to the mammalian environment is accompanied by extensive transcriptional remodelling in *Borrelia*, numerous studies have examined changes in gene expression during this transition, with the dominant *in vivo* approach being microarray analysis of *Borrelia* cultivated within dialysis membrane chambers (DMCs) implanted into rat peritoneal cavities. Across multiple such studies, alongside *in vitro* work, *BBJ23–BBJ28* are found to be up-regulated in conditions of mammalian infection, whereas *BBJ29* remains weakly expressed or undetectable. Both the increase in temperature and mammalian factors are triggers for these changes. For example, comparing temperature-shifted *in vitro* cultures to the rat model, it was possible to demonstrate that mammalian-specific factors, rather than temperature alone, were responsible for up-regulating the expression of *BBJ23* (1.85-fold), *BBJ26* (5.48-fold), and *BBJ27* (3.59-fold) [40]. In a separate study combing the effects of temperature and mammalian factors, *BBJ23* was found to be up-regulated 4.1-fold by comparing *in vitro* growth at 23°C, pH 7.5 (conditions of the unfed tick) to growth in DMCs in rats [41]. Similarly, *BBJ23* was up-regulated 5.1-fold when *Borrelia* were shifted from *in vitro* growth at 23°C, pH 7.5 (unfed tick) to *in vitro* growth at 37°C, pH 6.8 (fed tick) [41].

Evidence from the tick phase further supports regulated expression of the locus. *BBJ25* transcripts were not detected in unfed ticks by reverse-transcription polymerase chain reaction [42]. There was however a 12.3-fold up-regulation of *BBJ25* transcript as detected by cDNA microarray in response to a temperature shift and blood meal. The same study also discussed the possibility that *BBJ26, BBJ27*, and *BBJ28* form an operon and are components of an ABC transporter system, which is up-regulated in the mammalian host.

At the protein level, BBJ23, BBJ26, BBJ27, and BBJ28 were detected in whole cell lysate of wild-type *Borrelia* by tandem mass tag mass-spectrometry (TMT-MS) analyses, while BBJ24 protein was detected in a mutant strain of *bbd18* (a negative regulator of RpoS) but was not found in wild-type cells [43]. In this study, BBJ25 was only detected at the transcript level, not by TMT-MS.

Consistent with these microarray and proteomic results, the more recent RNA-Seq datasets have confirmed robust transcription across *BBJ23–BBJ28*, with a consistent absence of detectable transcripts corresponding to *BBJ29* [44].

One study that reports changes in all seven of these genes is an inactive mutant of the master transcriptional regulator Rrp2, which activates the RpoN–RpoS pathway, and is essential for the tick to mammal transition [45]. The expression of genes *BBJ23–BBJ29* was shown to be >3-fold higher in the wild type when compared to the *rrp2* mutant (ranges from 3.8- to 9.3-fold).

Taken together, the transcriptomic, proteomic, and regulatory evidence strongly supports the conclusion that *BBJ23–BBJ28* are up-regulated in the mammalian host, whereas *BBJ29* may be weakly expressed, or not expressed at all. Also consistent across these studies is a reduced level of expression of these genes in conditions replicating the unfed tick, which, when combined with the marked up-regulation in the mammalian host, indicates a role specific to the mammalian part of the lifecycle.

Throughout the *Borrelia* genus, the BBJ25 cluster is located on different linear genetic elements, either the smaller linear plasmids (lp38, lp17, lp28-3, lp28-8) or close to the end of the linear chromosome in the case of *B. turcica*. These smaller linear plasmids are relatively unstable, and it is well documented that there have been several inversions, recombination events, and lateral gene transfer events both within and between different *Borrelia* species [25]. The conservation of the complete cluster throughout the *Borrelia* genus possibly results from the selective advantage of regulating the stoichiometry of these proteins in response to the shift from arthropod to mammalian host.

Homologues of BBJ25 were found in all major families of *Spirochaetes* except for the *Leptospira* and *Brevinematales*, however the seven-gene cluster was only found in *Borrelia*, and so might reflect an adaptation to the two-host lifestyle. The conservation of BBJ25 in *Borrelia*, *Treponema*, and *Brachyspira*, a diverse range of spirochetes with different ecological niches and host–pathogen interactions suggests that BBJ25 performs a housekeeping role, rather than a direct role in pathogenicity such as host–pathogen interactions. Phylogenetic analysis of the branching order of the spirochetes suggests that the *Brachyspira* diverged prior to the emergence of the *Borrelia*, *Leptospira*, and *Treponema* lineages [46]. Therefore, the existence of BBJ25 in the *Brachyspira* implies a role for this protein in the last common ancestor of the *Spirochaetes*.

The Lol-sorting machinery of *E. coli* consists of five proteins LolABCDE. Although there is no equivalent to LolB in *Borrelia*, structural homologues were found to all other components including the periplasmic chaperone LolA and the ABC transporter LolCDE. *E. coli* LolCE is a heterodimeric permease, with LolE being primarily responsible for lipoprotein extraction from the IM [14]. Here, it is proposed that BBJ27 forms a homodimeric permease as occurs in the Lol-sorting machinery in some bacteria, e.g. LolF in *Francisella tularensis* [47]. BBJ26 is proposed to correspond to LolD, forming a dimer and harnessing energy from ATP hydrolysis to drive conformational changes in the permease domain (BBJ27) driving the extraction of a ligand from the IM. The AlphaFold3 model of BBJ28 has structural homology to *E. coli* LolA and, as it also has an SP1 signal sequence, is predicted to act as a periplasmic chaperone (Supplementary Figure S2).

A second LolA homologue has been identified in *Borrelia*, encoded on the chromosome, BB0346 has been shown to be involved in lipoprotein sorting [13]. Depletion of BB0346 led to mislocalisation of IM and periplasmic lipoproteins but did not influence surface-exposed lipoproteins. The X-ray structure of BB0346 [13] revealed the same β-half-barrel as predicted by AlphaFold3 for BBJ28, albeit with a central cavity occupied by PEG (Supplementary Figure S2). The coexistence of two LolA-like domains (BB0346 and BBJ28) is not unique to *Borrelia*. The Bacteroidota *Flavobacterium johnsoniae* has been shown to encode three LolA and two LolB proteins [48]. Furthermore, *E. coli* LolA and LolB are structural homologues, each with the same LolA-like open β-barrel topology and non-polar pocket. However, *E. coli* LolB is an OM lipoprotein, anchored to the OM, and is responsible for accepting lipoproteins from LolA in a mouth-to-mouth transfer and inserting them into the OM [49]. Consistent with previous studies, we failed to identify a direct homologue of the receptor lipoprotein LolB, therefore *Borrelia* likely has a different mechanism for inserting lipoproteins into the OM and controlling their surface exposure. In *Caulobacter vibrioides*, LolA functions as both a chaperone and insertase, enabling trafficking of lipoproteins directly to the OM despite the absence of LolB [50]. The mechanism of lipoprotein insertion in *Borrelia* could be similar, but with two LolA-like proteins (BB0346 and BBJ28). An alternative possibility is that BBJ25 acts as the OM-insertase.

## Future directions

Although further experimental work is required to confirm the subcellular localisation of these proteins, the functional predictions of BBJ23–BBJ28 are consistent with a role in the ATP-driven transport of a non-polar ligand from the IM to the OM. It is interesting to speculate on the nature of this ligand from the range of possible candidates (OM β-barrels, lipoproteins, glycolipids, sterols, etc.), some of which may be eliminated based on the existence of known transport systems. The export of OM β-barrel proteins is thought to be unlikely, as although BBJ25 is an Omp85 protein with structural homology to BamA/TamA, the absence of POTRA domains would preclude the transport of such large insoluble molecules across the periplasm. The transport of glycolipids likely occurs through the *Borrelia* LPT system [12]. However, the recent data are conflicting, in addition to transporting glycolipids the LPT system may also be involved in the transport of lipoproteins [12,15]. As suggested by others [13], it seems likely that there are multiple pathways for lipoprotein transport to the OM in *Borrelia*. The possibility that the BBJ23–28 cluster may play a role in the transport of lipoproteins and/or sterols cannot be ruled out, and further experimental work is required to investigate this.

## Supplementary Material

Supplementary Figures S1-S2 and Tables S1-S2

## Data Availability

All data to reproduce this work are available at https://github.com/RJBingham/Nixon_2025.

## Abbreviations

ABC: ATP-binding cassette
BAM: barrel assembly machinery
*B. burgdorferi*: *Borrelia burgdorferi*
*B. turcica*: *Borrelia turcica*
DALI: distance matrix alignment
DMC: dialysis membrane chamber
*E. coli*: *Escherichia coli*
IM: inner membrane
LD: Lyme disease
Lol: localisation of lipoprotein
OM: outer membrane
RF: relapsing fever
TMT-MS: tandem mass tag mass-spectrometry
TPR: tetratricopeptide repeat
SP1: signal peptidase type-1
