# Supplementary material for: Borrelia BBJ25 is a plasmid-encoded Omp85-family protein associated with a potential export system
Source: Biosci Rep. 2026 Feb 6;46(2):BSR20254082. doi: 10.1042/BSR20254082 (PMC13078400; doi:10.1042/BSR20254082)
Supplement: Supplementary Figures S1-S2 and Tables S1-S2 [file BSR-2025-4082_supp.pdf]

Supplementary Table 1 – List of complete non-redundant *Borrelia* Proteomes

		Protein Count	
**Proteome id**	**Subspecies**	**Chromosome**	**Plasmid**
UP000000611	Borrelia duttonii (strain Ly)	816	471
UP000000612	Borrelia recurrentis (strain A1)	799	178
UP000001205	Borrelia turicatae (strain 91E135)	818	206
UP000001634	Borrelia bissettiae (strain DSM 17990 / CIP 109136 / DN127)	816	587
UP000001807	Borreliella burgdorferi (strain ATCC 35210 / DSM 4680 / CIP 102532 / B31)	778	513
UP000002276	Borrelia garinii subsp. bavariensis (strain ATCC BAA-2496 / DSM 23469 / PBi)	832	430
UP000005212	Borrelia crocidurae (strain Achema)	864	605
UP000005216	Borreliella afzelii (strain PKo)	828	566
UP000006901	Borreliella burgdorferi (strain ZS7)	808	415
UP000008834	Borrelia hermsii (strain HS1 / DAH)	819	5
UP000019262	Borrelia anserina BA2	855	6
UP000019330	Borrelia coriaceae ATCC 43381	895	135
UP000019337	Borrelia crocidurae DOU	845	9
UP000075229	Borrelia hermsii	815	415
UP000264231	Borrelia turicatae	817	308
UP000275571	Borrelia turcica IST7	869	350
UP000291995	Borrelia miyamotoi	811	486
UP000326393	Borrelia maritima	802	131
UP000515603	Borrelia sp. A-FGy1	799	273
UP001164513	Borrelia miyamotoi	784	548
UP001164544	Borrelia miyamotoi	774	372

Supplementary Table 2. Summary of the Alphafold-DALI structure-function predictions. Alphafold3 models were subjected to a DALI search. The three highest scoring hits are shown with the domain, annotated function, PDB accession code and Z-score.

	Domain,<br>Function,<br>(PDB accession, Z-score)		
BBJ23	Tetratricopeptide repeat domain.<br>Sterol lipid transporter<br>(7T1S, 17.9)	Tetratricopeptide repeat domain.<br>Oligomer-forming<br>(2PL2, 17.3)	Tetratricopeptide repeat domain.<br>Lipoprotein transport<br>(4DI3, 16.9)
BBJ24	Tetratricopeptide repeat domain.<br>Oligomer-forming<br>(2PL2, 16.0)	Tetratricopeptide repeat domain.<br>Lipoprotein transport<br>(4DI3, 15.5)	Tetratricopeptide repeat domain.<br>Phospholipid binding<br>(7CC7, 15.2)
BBJ25	Omp85 superfamily domain (BamA/TamA)<br>Sorting and Assembly Machinery (SAM)<br>(7E4H, 17.6)	Omp85 superfamily domain (BamA/TamA)<br>Translocation and Assembly (TamA)<br>(8US3, 17.5)	Omp85 superfamily domain (BamA/TamA)<br>Barrel Assembly Machinery (BamA)<br>(8XNB, 17.3)
BBJ26	ABC transporter-like, ATP-binding domain<br>(3TUZ, 26.1)	ABC transporter-like, ATP-binding domain<br>(5GKO, 26.1)	ABC transporter-like, ATP-binding domain<br>(8JA7, 24.9)
BBJ27	MacB-like periplasmic core domain<br>Lipo-releasing system transmembrane protein LolE<br>(7MDY, 24.2)	MacB-like periplasmic core domain<br>Heme exporter<br>(7W78, 17.3)	MacB-like periplasmic core domain<br>MacB exporter<br>(5GKO, 16.2)
BBJ28	Lipoprotein localization factor, LolA-like<br>Unknown<br>(3BUU, 20.2)	Lipoprotein localization factor, LolA-like<br>DUF1329<br>(4Z48, 18.4)	Outer membrane lipoprotein carrier protein LolA<br>Lipoprotein carrier<br>(8CGM, 12.7)
BBJ29	Porin<br>OM carboxylate channel<br>(5DL7, 21.7)	Porin<br>OM carboxylate channel<br>(5DL5, 21.6)	Porin<br>Chitoporin<br>(7VTZ, 20.8)

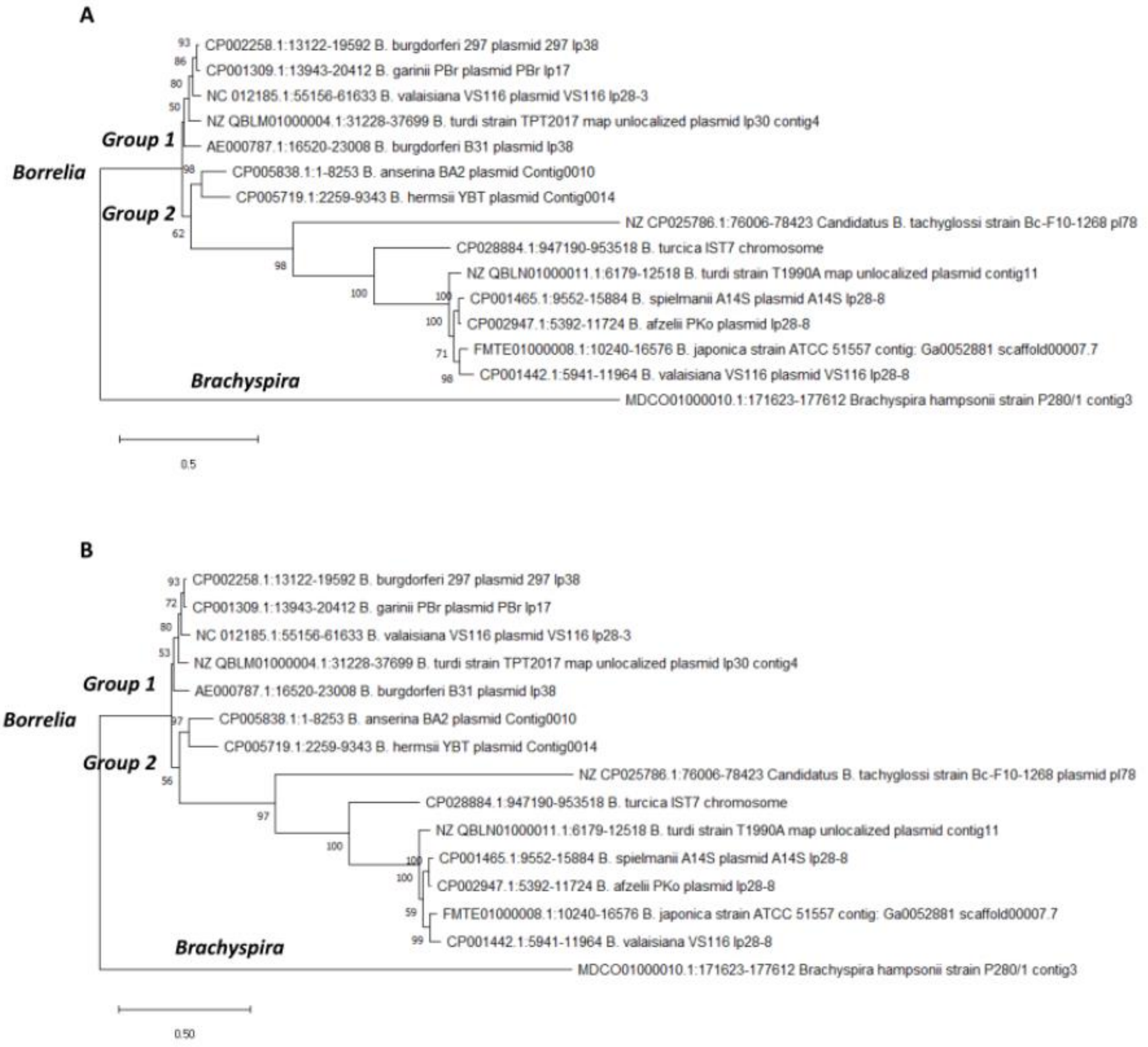

Supplementary Figure 1. Phylogenetic analysis of predicted BBJ25 operon DNA sequences from *Borrelia* species and *Brachyspira*. A) Analysis of the whole operon based on genomic regions shown with 10158 positions. B) Analysis of the equivalent regions with omission of the BBJ25 coding sequence with a total of 7845 positions. DNA sequences were aligned using MUSCLE [33]. The evolutionary history was inferred by using the Maximum Likelihood method and General Time Reversible model. The percentage of trees in which the associated taxa clustered together is shown next to the branches based on 1000 bootstrap replications. The tree is drawn to scale, with branch lengths measured in the number of substitutions per site. This analysis involved 15 nucleotide sequences. Evolutionary analyses were conducted in MEGA X [34]

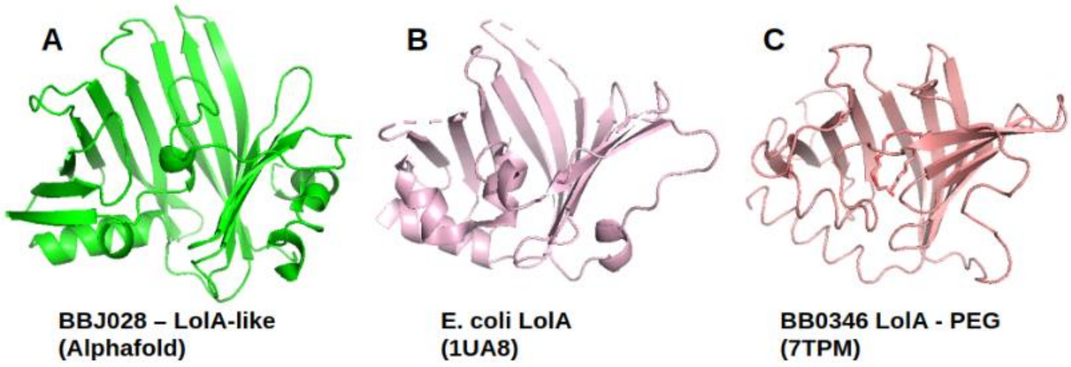

Supplementary Figure 2. Borrelia BBJ28 and BB0346 are both LolA-like proteins. The Alphafold3 model of BBJ28 (A) and the X-ray structure of E. coli LolA (B) both have an open beta barrel fold with a non-polar central cavity occupied by a short alpha helix. The X-ray structure of BB0346 (C) reveals a similar fold but the helix has been displaced by PEG.

Pairwise structural alignment was conducted using DALI, The RMSD between BBJ28 and *E. coli* LolA is 3.2 Å, while the RMSD between BB0346 and *E. coli* LolA (1UA8) is 3.6 Å Ang over 155 residues. The higher RMSD is probably related to the bound PEG displacing the central helix.
